# Postural Sway Characteristics in Patients With Persistent Postural-Perceptual Dizziness: A Comparative Study Between Psychogenic Vertigo Patients and Healthy Controls

**DOI:** 10.7759/cureus.82597

**Published:** 2025-04-19

**Authors:** Toru Miwa

**Affiliations:** 1 Otolaryngology, Teikyo University Hospital, Kanagawa, JPN

**Keywords:** persistent postural-perceptual dizziness, primary pppd, psychogenic vertigo, secondary pppd, static posturography

## Abstract

This study aimed to compare postural sway characteristics and evaluate postural sway as a potential biomarker for persistent postural-perceptual dizziness (PPPD) to clarify diagnostic boundaries. A total of 36 participants were enrolled, including 26 (72.2%) with PPPD, five (13.9%) with psychogenic vertigo, and five (13.9%) healthy controls. Postural sway tests were conducted on all participants, measuring the center of pressure (CoP) area, CoP length, and Romberg ratio. A subgroup analysis was also performed based on the presence of preceding organic diseases. The postural sway tests were conducted using a force platform under standardized conditions. Each participant stood barefoot on a stable surface for 60 seconds with eyes open and then with eyes closed, while data were sampled at 100 Hz. The results showed that both the PPPD and psychogenic vertigo groups exhibited significantly larger CoP areas compared to healthy controls, indicating increased postural instability. However, no significant difference was found between the PPPD and psychogenic vertigo groups. While CoP length in the PPPD group was similar to that of healthy controls, the psychogenic vertigo group showed a significantly greater CoP length with eyes closed. These findings suggest that patients with PPPD maintain fine postural control despite increased sway, indicating preserved central nervous system adaptation. No differences were observed between primary and secondary PPPD (s-PPPD). Overall, the postural sway characteristics of PPPD closely resembled those of psychogenic vertigo, supporting the notion that both conditions share features of functional dizziness disorders. Future research should include larger, age- and sex-matched cohorts, along with advanced posturography methods and detailed symptom assessments, to refine diagnostic criteria.

## Introduction

Persistent postural-perceptual dizziness (PPPD) is a functional vestibular disorder first defined by the Bárány Society in 2017 [1]. PPPD is characterized by chronic dizziness persisting for at least three months, with non-rotational vertigo, floating sensations, and postural instability. These symptoms are typically exacerbated by standing, walking, active or passive body movements, and exposure to complex visual stimuli [1]. The underlying mechanism of PPPD is thought to involve a disruption in multisensory integration following a vestibular, visual, or somatosensory disturbance caused by a preceding event. This disruption results in inappropriate sensory reweighting, where excessive reliance on visual input occurs at the expense of vestibular and somatosensory information [2-4]. This maladaptive shift toward visual dependency and increased sensitivity to minor visual or motion stimuli contributes to the persistence of dizziness and postural instability, even after the initial triggering condition resolves [5].

Currently, the diagnosis of PPPD relies primarily on clinical interviews, supplemented by tools such as the Niigata PPPD Questionnaire (NPQ), which assesses factors that exacerbate symptoms [6]. However, there are no established biomarkers or objective diagnostic tests, such as imaging or vestibular function assessments, that reliably identify PPPD [1]. This diagnostic gap has prompted the exploration of alternative methods, including postural sway analysis, to identify characteristic patterns of postural instability and sensory weighting abnormalities in patients with PPPD. Notably, Ichijo et al. [7] proposed that postural sway parameters obtained under conditions involving foam rubber surfaces could reflect increased visual dependence and reduced somatosensory reliance, potentially distinguishing PPPD from other vestibular disorders.

In clinical practice, distinguishing patients with PPPD from those with psychogenic vertigo can be particularly challenging. Psychogenic vertigo, while sharing some symptomatic overlap with PPPD, arises from psychological mechanisms such as anxiety, somatization, or hypervigilance, rather than a primary dysfunction in sensory integration [8]. “Sensory weighting” refers to the process by which the brain assigns varying levels of importance to visual, vestibular, and somatosensory inputs to maintain balance. “Functional dizziness” is a group of disorders in which dizziness persists despite the absence of structural or neuro-otologic abnormalities, often linked to psychological or behavioral factors.

This study aimed to compare postural sway characteristics in patients with PPPD, psychogenic vertigo, and healthy controls to determine whether sway patterns can serve as potential biomarkers. We hypothesized that PPPD patients would demonstrate greater visual dependence than those with psychogenic vertigo. Additionally, we examined whether preceding organic vs. non-organic conditions influence postural sway within the PPPD group [9]. Clarifying these differences may help improve diagnostic accuracy and understanding of underlying mechanisms.

## Materials and methods

This study was designed as a retrospective cohort study involving patients who attended the Department of Otolaryngology at Osaka Metropolitan University Hospital between January 2021 and April 2023. Twenty-six patients were diagnosed with PPPD based on the diagnostic criteria established by the Bárány Society [1]. In addition, five patients diagnosed with psychogenic vertigo during the same period were included as a comparison group [8]. To establish a baseline reference, five healthy volunteers from the Osaka Metropolitan University School of Medicine were also recruited. This study was approved by the Institutional Review Board of Osaka Metropolitan University (Approval No.: 2020-082). This study was conducted in accordance with the ethical principles outlined in the Declaration of Helsinki. In this retrospective study, informed consent was waived by the IRB due to the use of anonymized data.

Inclusion and exclusion criteria　

Inclusion criteria required that participants be aged 18 years or older, be capable of maintaining a standing position with their eyes closed for at least 60 seconds, and be attending their first consultation for dizziness at the clinic, regardless of the timing of symptom onset, severity, or recurrence. Exclusion criteria included patients whose dizziness symptoms had fully resolved by the time of the visit and patients who declined to participate in the study.

Postural sway testing

All participants underwent static posturography using the GP-31 stabilometer (Anima, Tokyo, Japan) without the use of foam rubber according to the protocol recommended by the Japan Society for Equilibrium Research [10]. A few participants underwent testing using foam rubber, but the results of the foam rubber tests were not used because the data from all participants was to be used. Each participant stood upright on the force plate with feet together and arms at their sides for 60 seconds under two conditions: with eyes open and with eyes closed. The subject is asked to stand facing a specified direction with the inner edges of both feet touching the center of the post of the postural sway meter and to look at the visual target placed 2 to 3 meters in front of them. Force plate calibration was performed before each session, and data were sampled at 100 Hz. The system measured both the center of pressure (CoP) area and CoP length in each condition. The examiner was blinded to participant diagnoses to minimize assessment bias.

Symptom assessment

Participants were asked to complete the Dizziness Handicap Inventory (DHI), a validated tool for quantifying the impact of dizziness on daily activities [11]. For healthy subjects, history-taking confirmed the absence of dizziness, but no formal screening tools were used.

Calculation of Romberg ratio

The Romberg ratio, a common indicator of visual dependence, was calculated for both CoP area and CoP length by dividing the eyes-closed measurement by the eyes-open measurement.

Subgroup analysis within PPPD

Following the approach described by Habs et al. [9], patients with PPPD were categorized into two subgroups based on the nature of their preceding conditions. Patients with preceding non-organic conditions (e.g., psychiatric disorders) were classified as having primary PPPD (p-PPPD), while those with preceding organic vestibular disorders (e.g., benign paroxysmal positional vertigo (BPPV), vestibular neuritis, or Meniere’s disease) were classified as having secondary PPPD (s-PPPD). Both CoP area and CoP length were compared between these subgroups.

Statistical analysis

Statistical comparisons between groups were conducted using a one-way analysis of variance (ANOVA) followed by Tukey’s post hoc test. Differences in gender distribution were analyzed using Fisher’s exact test. Effect sizes (w or R2 where applicable) were calculated to evaluate the magnitude of group differences. Statistical significance was set at p < 0.05. All analyses were performed using GraphPad Prism (version 10.2.3; GraphPad Software, San Diego, CA, USA). Due to the small sample size and group imbalance in age and sex, statistical adjustments were not feasible and were acknowledged as a limitation.

## Results

Participant characteristics

Table 1 presents the demographic and clinical characteristics of the three groups. The healthy control group was significantly younger than both the PPPD group and the psychogenic vertigo group (p < 0.001, R2 = 0.75, p < 0.001), and the gender distribution also differed significantly between groups (p = 0.01, w = 0.71, p = 0.05). As expected, DHI scores were significantly lower in the healthy control group than in both patient groups (p < 0.001, R2 = 0.61, p < 0.001). Among patients with PPPD, 10 (38.5%) had p-PPPD, and 16 (61.5%) had s-PPPD. Among those classified as p-PPPD, 10 (100%) had psychogenic vertigo. Among those classified as s-PPPD, 11 (68.75%) had BPPV, one (6.25%) had Meniere’s disease, two (12.5%) had vestibular neuritis, one (6.25%) had Hunt syndrome, and one (6.25%) had motion sickness.

**Table 1:** Demographic and Clinical Characteristics of Each Group ^a^One-way ANOVA with post hoc Tukey test ^b^Fisher's exact test *p < 0.05 ***p < 0.001 Data are presented as mean (range). PPPD, persistent postural-perceptual dizziness; DHI: Dizziness Handicap Inventory

	Healthy	PPPD	Psychogenic vertigo	p-value
Number of cases	5	26	5	
Age (years)	23.0 (22-24)	54.4 (28-82)	67.4 (48-80)	<0.001***^a^
Sex (male:female)	4:1	6:20	0:5	0.01*^b^
DHI score	0.4 (0-2)	49.2 (18-92)	86.4 (78-90)	<0.001***^a^

Postural sway analysis

CoP Area

**Figure 1:**
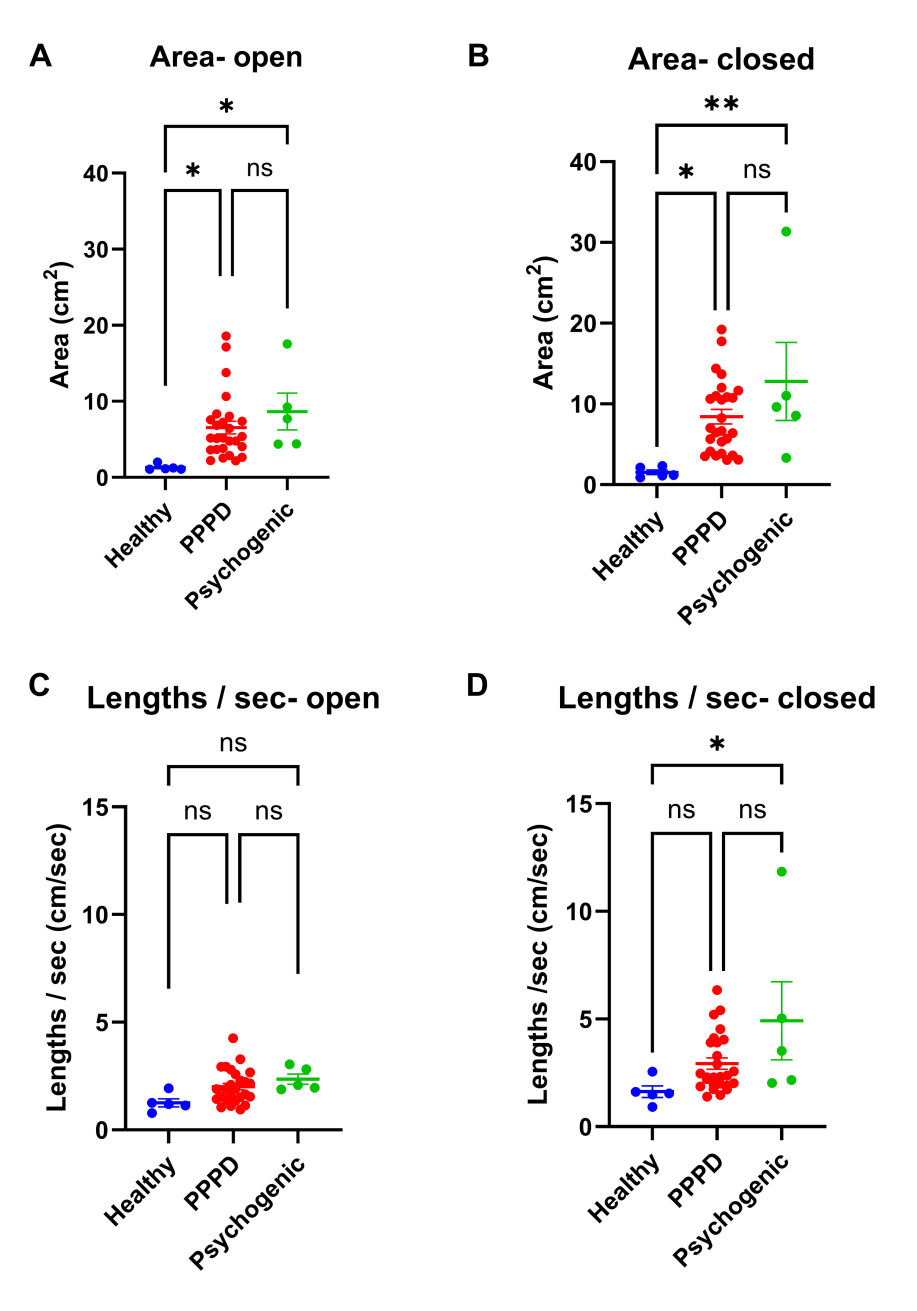
Comparison of CoP Area and CoP Length Comparison of CoP area under eyes-open (A) and eyes-closed (B) conditions. Comparison of CoP length under eyes-open (C) and eyes-closed (D) conditions. *p < 0.05 **p < 0.01 CoP, center of pressure; PPPD, persistent postural-perceptual dizziness; ns, not significant

Both the PPPD group and the psychogenic vertigo group demonstrated significantly larger CoP areas than the healthy control group in both the eyes-open condition (Figure 1A, PPPD: 6.54 cm²; psychogenic vertigo: 8.66 cm²; healthy controls: 1.31 cm²; p = 0.04 and p = 0.02, respectively, R2 = 0.20, p = 0.02) and the eyes-closed condition (Figure 1B, PPPD: 8.40 cm²; psychogenic vertigo: 12.7 cm²; healthy controls: 1.52 cm²; p = 0.04 and p = 0.007, respectively, R2 = 0.24, p = 0.009). However, no significant differences in CoP area were observed between the PPPD and psychogenic vertigo groups (Figure 1A; Figure 1B).

CoP Length

In the eyes-open condition, no significant differences in CoP length were found between any of the three groups (Figure 1C). However, in the eyes-closed condition, CoP length was significantly larger in the psychogenic vertigo group than in the healthy control group (Figure 1D, p = 0.02, R2 = 0.19, p = 0.03). No significant differences were found between the PPPD group and the psychogenic vertigo group (Figure 1C; Figure 1D).

Romberg Ratio

**Figure 2:**
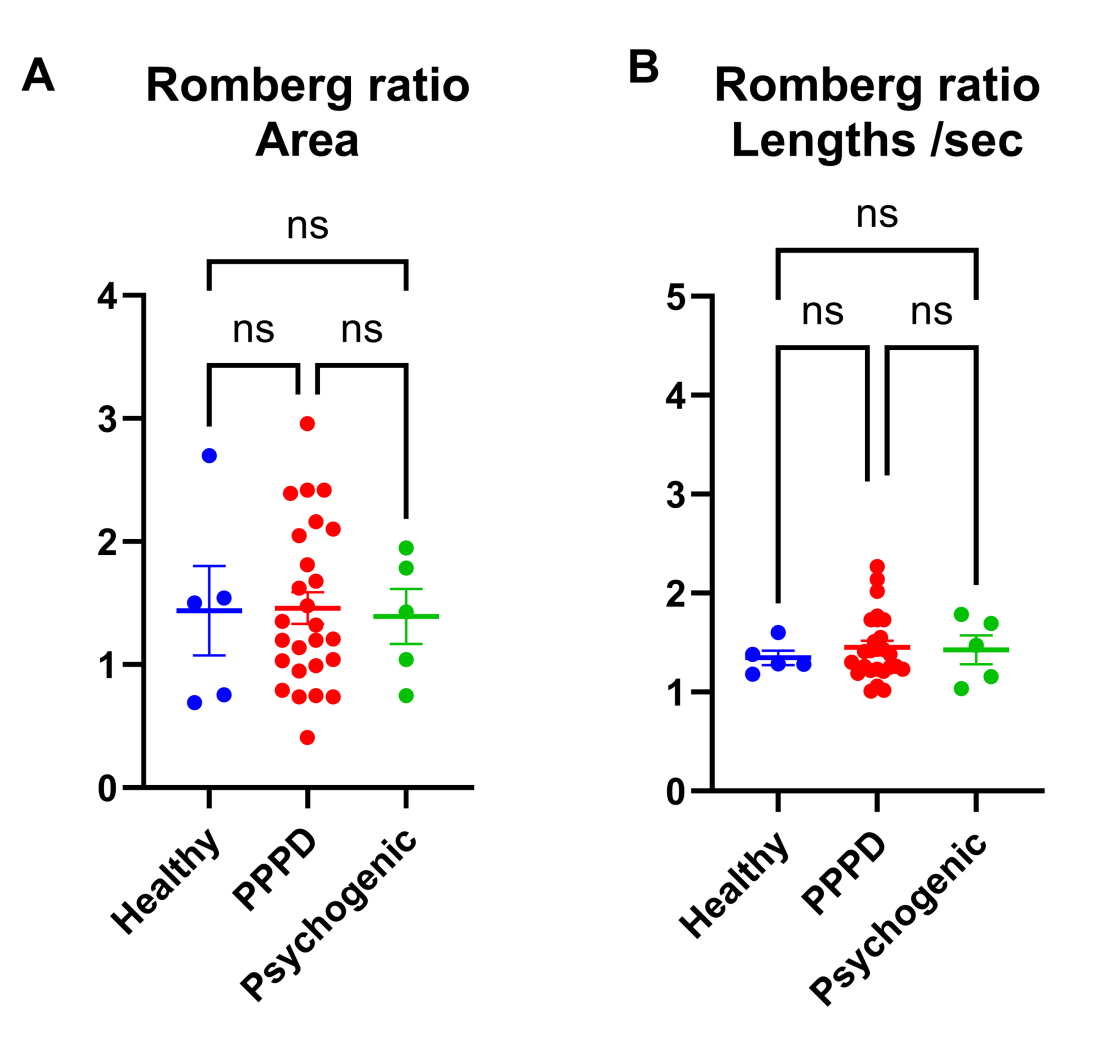
Comparison of Romberg Ratios for CoP Area and CoP Length (A, B) Comparison of Romberg ratios for CoP area (A) and CoP length (B) across groups. CoP, center of pressure; PPPD, persistent postural-perceptual dizziness; ns, not significant

Romberg ratios for both CoP area and CoP length did not significantly differ among the three groups (Figure 2A; Figure 2B).

Subgroup analysis

P-PPPD vs. S-PPPD

**Figure 3:**
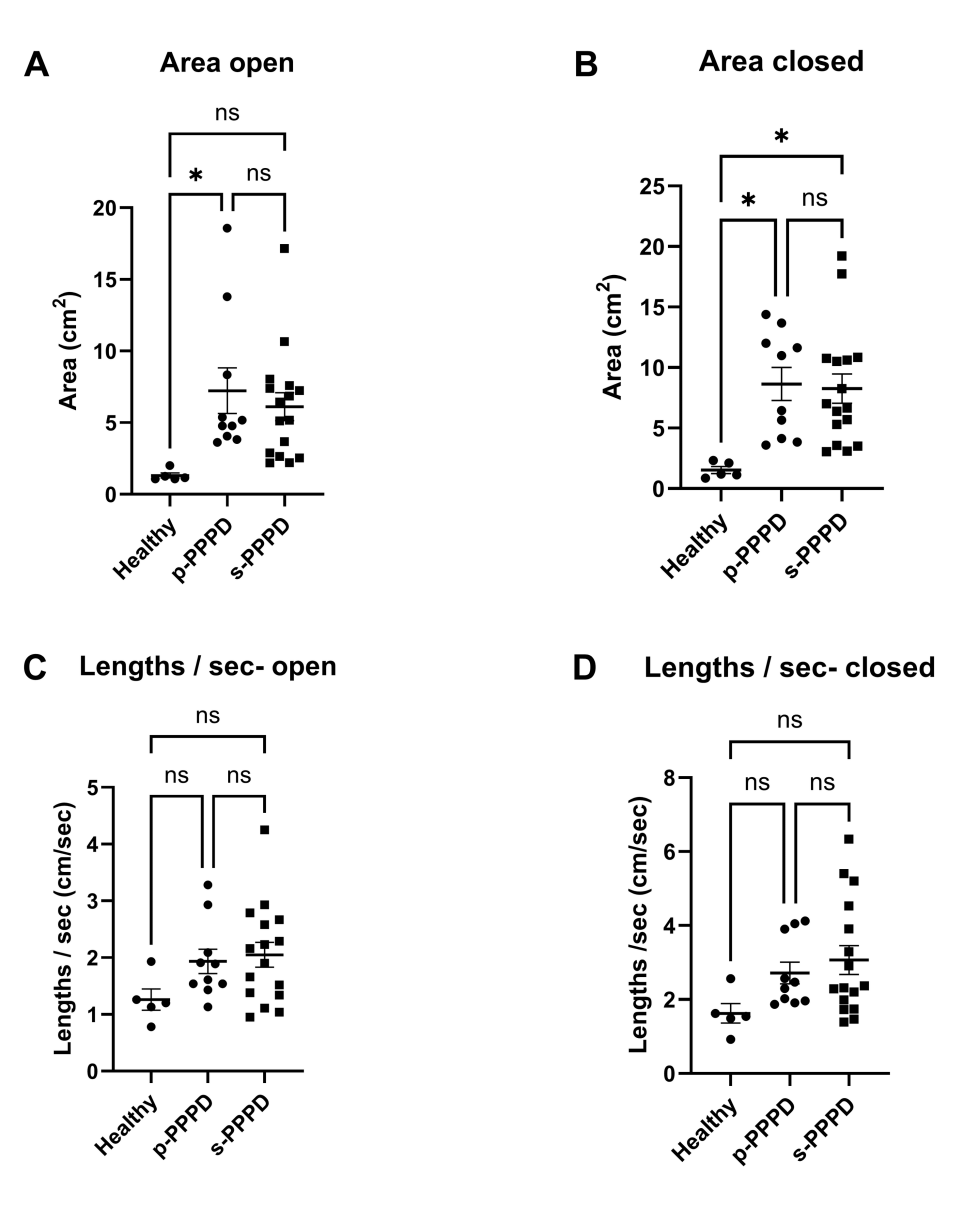
Comparison of CoP Area and CoP Length by Preceding Conditions in PPPD Comparison of CoP area under eyes-open (A) and eyes-closed (B) conditions among p-PPPD, s-PPPD, and healthy groups. Comparison of CoP length under eyes-open (C) and eyes-closed (D) conditions among p-PPPD, s-PPPD, and healthy groups. *p < 0.05 CoP, center of pressure; PPPD, persistent postural-perceptual dizziness, p-PPPD, primary PPPD; s-PPPD, secondary PPPD; ns, not significant

Comparison of CoP area within the PPPD group showed that both p-PPPD and s-PPPD patients had significantly larger CoP areas than those of healthy controls in both the eyes-open (Figure 3A, p = 0.03, R2 = 0.21, p = 0.04) and eyes-closed (Figure 3B, p = 0.01, R2 = 0.27, p = 0.01) conditions. However, there were no significant differences between p-PPPD and s-PPPD, nor were there any significant differences in CoP length between these two subgroups (Figure 3C; Figure 3D).

## Discussion

This study investigated differences in postural sway between patients with PPPD, psychogenic vertigo, and healthy controls. Our results revealed that both the PPPD group and the psychogenic vertigo group exhibited significantly larger CoP areas than the healthy control group, indicating greater postural sway in both patient populations. However, no significant difference was found between the PPPD group and the psychogenic vertigo group in terms of CoP area, suggesting that these two conditions share similar patterns of instability in postural control. In contrast, CoP length, a parameter reflecting the precision of postural adjustments [12], was significantly larger in the psychogenic vertigo group than in the healthy control group when the eyes were closed, while no significant difference in CoP length was found between the PPPD group and the healthy control group. This finding implies that, despite greater overall sway (larger CoP area), patients with PPPD maintained postural control precision comparable to that of healthy individuals, suggesting that the central nervous system’s sensory integration and adaptive strategies remain largely preserved in PPPD.

The observed differences between the PPPD and psychogenic vertigo groups with regard to CoP length may reflect differences in central sensory processing and motor control efficiency. However, both groups ultimately demonstrated similarly increased instability relative to healthy controls. The similarities in sway patterns between PPPD and psychogenic vertigo suggest that both conditions may share common pathophysiological features characteristic of functional dizziness disorders, as proposed in previous literature [1]. This overlap is consistent with prior work indicating that both conditions are linked to maladaptive sensory weighting, increased visual dependence, and heightened sensitivity to both internal and external stimuli [2-5].

The weighting hypothesis, which suggests that PPPD results from inappropriate central weighting of vestibular, visual, and somatosensory inputs following a vestibular event, has been proposed as a central mechanism underlying PPPD [1,2]. This hypothesis is further supported by functional imaging studies showing altered responses in the insula and occipital cortex in patients with PPPD, regions implicated in sensory integration and spatial orientation [4]. Consistent with this hypothesis, Ichijo et al. [7] demonstrated that patients with PPPD show increased visual dependence and reduced somatosensory dependence during foam-based posturography, further highlighting their altered sensory weighting process. Similarly, McCaslin et al. [13] reported that patients with PPPD exhibited greater postural sway during wearable sensor-based testing, particularly under visually challenging conditions, supporting the notion that these patients rely excessively on visual input for balance.

Interestingly, our findings diverge from prior studies that identified clear differences in visual dependence between patients with PPPD and healthy controls using foam-based posturography [7,13,14]. In our study, the Romberg ratio, a commonly used indicator of visual dependence, showed no significant differences between groups. This discrepancy could be explained by the absence of foam rubber conditions, which are commonly used to suppress somatosensory input and enhance the detection of visual dependence [7,14,15]. Somatosensory inputs were likely preserved in our study, potentially masking typical visual dependence effects in PPPD. Given these findings, it is possible that the visual dependence commonly associated with PPPD was underestimated due to methodological constraints in this study.

Further complicating interpretation, PPPD is a heterogeneous disorder with varied clinical presentations depending on the nature of the initial precipitating event. Habs et al. [9] emphasized the importance of distinguishing between p-PPPD and s-PPPD. Our subgroup analysis found no significant differences between p-PPPD and s-PPPD in terms of CoP area or CoP length. This may suggest that postural control disturbance in PPPD is independent of the nature of the preceding event, but more data are needed to support this conclusion.

The findings in the psychogenic vertigo group are also noteworthy. Unlike patients with PPPD, they demonstrated increased CoP length when their eyes were closed. This pattern may reflect more generalized motor dysregulation, affecting both balance stability and postural control precision. These findings are consistent with literature suggesting that psychogenic vertigo may involve central disturbances in motor planning and proprioception [16].

These results must be interpreted in light of several limitations. First, the small sample size raises concerns about statistical power and may have limited our ability to detect true group differences. Second, the retrospective nature of the study introduces potential selection bias. Third, the comparison groups were not age- and sex-matched, and the small sample size made statistical correction unfeasible. Age-related changes in balance control and postural sway are well-documented and may have contributed to group differences [14]. Fourth, the diagnostic overlap between p-PPPD and psychogenic vertigo complicates interpretation, as all p-PPPD cases could be classified as psychogenic vertigo. Fifth, many potential participants were excluded due to a lack of NPQ administration or posturography with foam rubber, potentially limiting generalizability. Finally, unmeasured confounding variables (e.g., anxiety and medication use) may have influenced postural sway patterns. Future prospective studies with larger, matched samples and standardized assessment protocols are needed to further clarify the diagnostic utility of postural sway analysis in functional dizziness disorders.

## Conclusions

Our findings support the view that PPPD and psychogenic vertigo share certain characteristics of postural instability, particularly increased postural sway. However, patients with PPPD retain fine postural control, indicating preserved central nervous system integration and adaptation mechanisms, which may differentiate them from patients with psychogenic vertigo. These findings reinforce the importance of understanding PPPD as a disorder of altered sensory integration weighting rather than a condition purely related to psychogenic factors or motor control dysfunction.

Clinically, this highlights the importance of distinguishing PPPD from psychogenic vertigo to guide appropriate therapeutic strategies, including vestibular rehabilitation and psychological support tailored to sensory integration deficits. Future research should focus on accumulating a larger number of cases to improve the generalizability of findings and incorporate foam rubber posturography to better assess subtle balance control characteristics. In addition, comprehensive symptom assessment tools, such as the NPQ, should be used to clarify symptom patterns and contribute to more precise diagnostic criteria.
